# Donor-derived mycosis fungoides following reduced intensity haematopoietic stem cell transplantation from a matched unrelated donor

**DOI:** 10.1136/bcr-2016-216331

**Published:** 2017-01-10

**Authors:** Francesca A M Kinsella, Mohammad Rasoul Amel Kashipaz, Julia Scarisbrick, Ram Malladi

**Affiliations:** 1 Institute of Immunology and Immunotherapy, University of Birmingham, Birmingham, UK; 2 Department of Cellular Pathology, University Hospital Birmingham NHS Foundation Trust, Birmingham, UK; 3 Department of Dermatology, University Hospital Birmingham NHS Foundation Trust, Birmingham, UK

## Abstract

A 46-year-old woman with a history of dasatinib-resistant chronic myeloid leukaemia, clonal evolution and monosomy 7 underwent reduced intensity conditioned in vivo T-cell-depleted allogeneic haematopoietic stem cell transplantation (HSCT) from a matched unrelated donor. Following the transplantation, she developed recurrent cutaneous graft versus host disease (GvHD), which required treatment with systemic immunosuppression and electrocorporeal photophoresis. Concurrently, she developed a lichenoid rash with granulomatous features suggestive of cutaneous sarcoidosis. Additional treatment with hydroxychloroquine was initially successful, but 2 months later, she developed erythroderma with palpable lymphadenopathy. Repeated histological analysis established a diagnosis of folliculotropic mycosis fungoides stage IVA2, and the malignant clone was confirmed to be of donor origin. A positive response to brentuximab has been shown. This is the first reported case of primary mycosis fungoides after matched unrelated donor HSCT, and in a patient still undergoing treatment for GvHD.

## Background

Allogeneic haematopoietic stem cell transplantation (HSCT) was the standard of care for patients for chronic myeloid leukaemia (CML) until the advent of tyrosine kinase inhibition. Since then, HSCT has been reserved for those with resistance to tyrosine kinase inhibition or advanced disease.^1^ HSCT is associated with an increased risk of secondary malignancies, the most common of which are post-transplant lymphoproliferative disorders (PTLD). Long-term immunosuppression with calcineurin inhibitors such as ciclosporin and tacrolimus is thought to contribute to this increased risk, as described in cardiac transplantation where profound immunosuppression is required within the first year.^2^ PTLD after HSCT is usually comprised of Epstein Barr Virus (EBV)-associated donor-derived B-cell lymphomas, which typically arise in the first 6 months post-HSCT.^3^ In contrast, T-cell lymphomas are rarer and are not EBV associated. While up to 15% of PTLD after solid organ transplantation are T-cell derived, there are only anecdotal reports of T-cell lymphoma following HSCT.^4–9^ Here, we report what is to the best of our knowledge, the first case of mycosis fungoides following HSCT from a HLA-matched unrelated donor. Despite its rarity, this case exemplifies the need for a high index of suspicion in patients with indistinct clinical pathology who have risk factors for secondary malignancies, particularly prolonged and profound immunosuppression. Serial histopathological, molecular analyses and the expertise of three disciplines were required to reach a diagnosis. A positive response to brentuximab has been shown.

## Case presentation

A 46-year-old woman with a history of CML diagnosed in the first chronic phase demonstrated dasatinib resistance associated with clonal evolution and monosomy 7. Consequently, she underwent a reduced intensity conditioned (fludarabine 30 mg/m^2^ for 5 days and melphalan 140 mg/m^2^ for 1 day) in vivo T-cell-depleted (alemtuzumab total dose—50 mg) unrelated donor allogeneic haematopoietic stem cell transplant (HSCT) in August 2010. Her donor was a man and matched for the human leucocyte antigens (HLA) A, B, C, DRB and DRQ. Graft versus host disease (GvHD) prophylaxis with ciclosporin was initiated 2 days pre-transplant.

At day 100 post-transplant, examination of bone marrow revealed complete donor chimerism and a morphological remission. *BCR-ABL* transcripts have remained undetectable since so that it is likely a cure was achieved.

**Figure 1:**
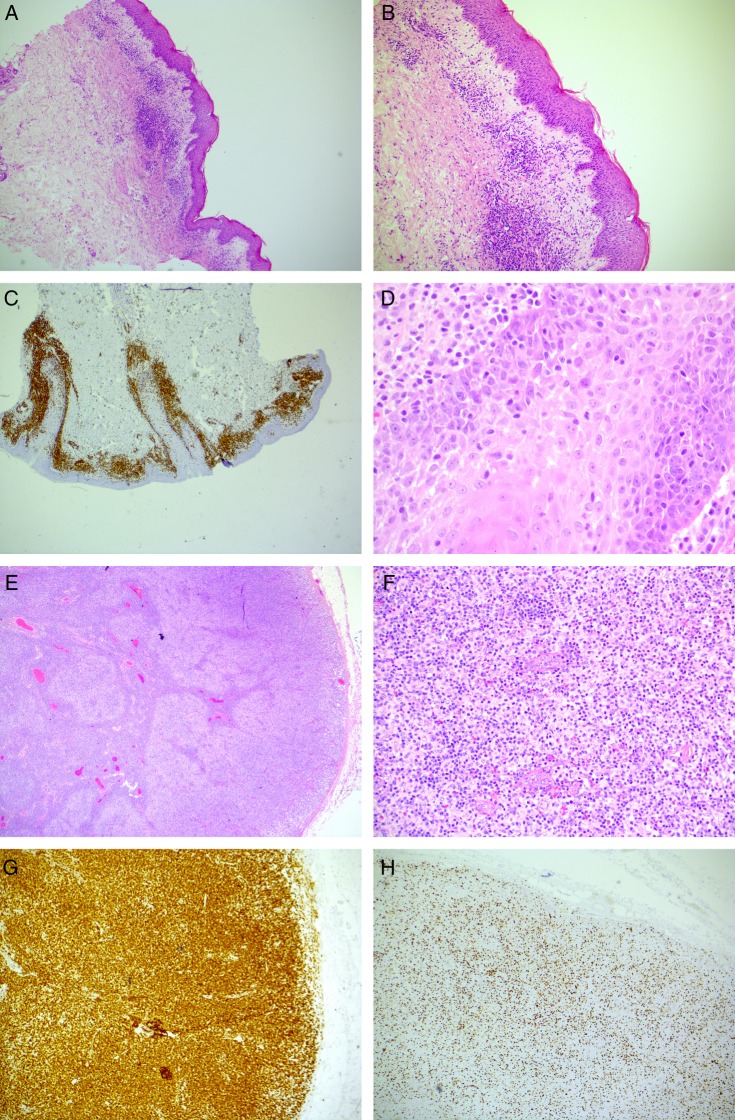
Skin biopsy histology. (A) October 2010. Skin biopsy of the initial diagnostic rash of acute GvHD (×10). (B) November 2013. Skin biopsy of the progressive rash involving epidermis, dermis and subcutis with dense per-vascular lymphocytic infiltrate (×4). (C) November 2014. Skin biopsy of the erythrodermic rash revealed extensive CD3 expression denoting a T-lymphocytic infiltrate (×20). (D) The skin infiltrate from was made up of atypical T-cells with nuclear irregularity and pale cytoplasm (×600). (E). Excised peripheral draining lymph node had a disrupted follicular architecture (×20). (F) The follicular architecture was effaced by atypical lymphocytes similar to those found in the skin (×200). (G). The atypical lymphocytes effacing the lymph node follicular region were CD3+ T-cells (×40). (H) Approximately 30% of the lymph node T-cell infiltrates expressed Ki-67 (×40).

In October 2010, the patient developed biopsy-proven acute cutaneous GvHD (figure 1A). This was treated with oral prednisolone and continued ciclosporin, but she developed recurrent severe cutaneous chronic GvHD and was unable to withdraw from oral immunosuppression. Thus, extracorporeal photopheresis (ECP) was started in March 2012, and over the next 20 months, immunosuppression was tapered to 10 mg prednisolone daily and 150 mg ciclosporin daily.

In November 2013, the patient developed a new pruritic and lichenoid rash atypical of the preceding GvHD (figure 1B) consistent with cutaneous sarcoidosis. This rash initially responded to treatment with hydroxychloroquine in conjunction with continued immunosuppression and ECP, but in July 2014, the patient developed a worsening of the rash with intense pruritus and erythroderma. A trial of drug withdrawals did not improve the eruption, which continued to evolve. By November 2014, it was accompanied by palpable cervical lymphadenopathy.

## Investigations

Investigations were carried out in November 2014.

fluorodeoxyglucose positron emission tomograghy (FDG-PET) imaging revealed lymphadenopathy above and below the diaphragm. The largest lymph nodes measured up to 3.2×1.7 cm were located in the pre-pectoral and axillary regions, and were the most avid (standardised uptake value (SUV) max 5.8). Histological examination of the skin at this point revealed a loss of features characteristic for GvHD or granulomatous infiltrate, but displayed an atypical T-lymphocytic infiltrate (figure 1C, D) with folliculotropism. Histological analysis of an enlarged cervical lymph node revealed complete effacement of nodal architecture by small atypical lymphocytes with marked nuclear irregularity and pale cytoplasm (figure 1E, F). The T-cell infiltrate displayed an inverted CD4:CD8 ratio of 1:2, and immunohistochemical studies showed that the infiltrate expressed CD3 (figure 1G), CD4, CD5 and CD43. Thirty per cent had downregulated CD7 surface expression, while 30% expressed Ki67 (figure 1H), and the infiltrate was Epstein Barr virus encoded RNAs (EBER) negative.

**Figure 2:**
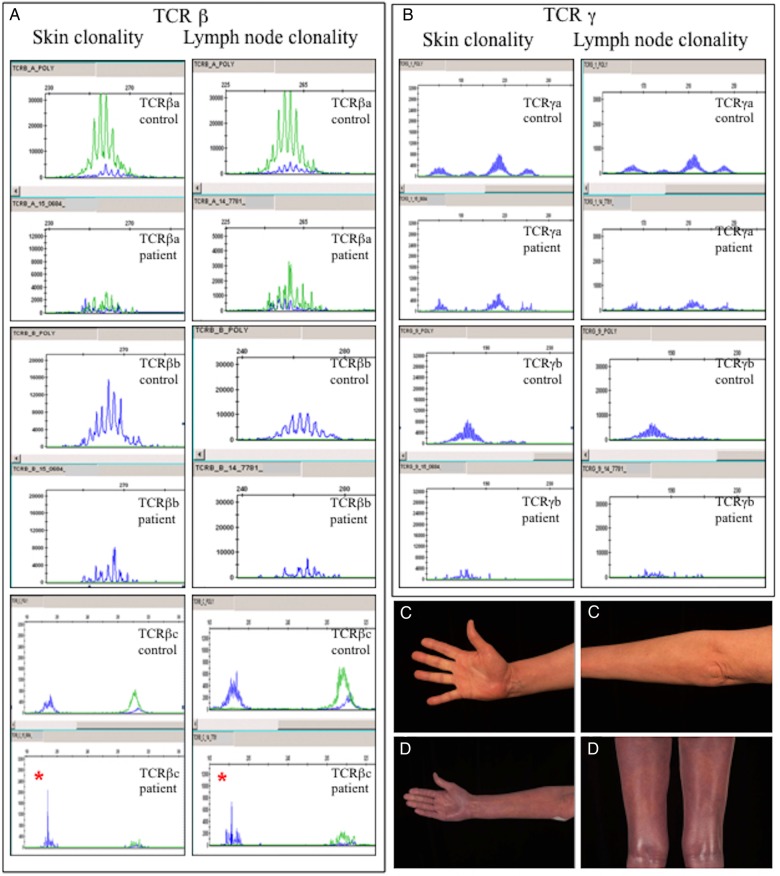
(A) TCRβ gene clonality. Results from the skin and lymph node were obtained 4 years post-RIC HSCT. Weak clonality of the TCRβc region * and a restricted repertoire of the TCRβb region were found in skin and lymph node. (B) TCRγ gene clonality. The TCRγ gene locus was polyclonal. (C) Clinical photography taken of areas of the skin affected by erythroderma at the time of diagnosis of CTCL (4 years post-HSCT), prior to treatment, and (D) following the failure of gemcitabine mono therapy.

Clonality studies confirmed the presence of an identical clonal TCRβ rearrangement in skin and lymph node biopsies, thus confirming a diagnosis of cutaneous T-cell lymphoma (CTCL) (figure 2A, B), stage IVA2 (T4 N3 M0 B0) folliculotropic mycosis fungoides (T4, N3, M0, B0) (figure 2C) and stage N3 according to the EORTC WHO classification.^10^

Microsatellite and XY-FISH studies confirmed that the malignant infiltrate was of donor origin.

## Treatment

Please see the ‘Outcome and follow-up’ section.

## Outcome and follow-up

The patient remained erythematous (mSWAT score of 200) and failed to respond to 4 cycles of gemcitabine monotherapy and taper of immunosuppression. Further FDG-PET imaging revealed an increase in metabolic activity of affected nodes (axillary lymph node SUV max 6.9), along with new splenomegaly (24 cm) exhibiting increased diffuse activity. While still receiving ECP for GvHD, she initiated brentuximab (1.8 mg/kg dose) with the aim of disease control. After 3 cycles, she experienced significant diarrhoea and grade 1 peripheral neuropathy, and the dose of brentuximab was reduced to 1.2 mg/kg. She has subsequently received a further four cycles with no further detrimental effects, and has experienced a significant reduction in lymphadenopathy and B symptoms. Her quality of life has improved, and her Eastern Cooperative Oncology Group (ECOG) has dropped from 3 to 1. However, despite these improvements, she remains pruritic with an mSWAT score of 100 (figure 2D).

## Discussion

We report the first case of erythrodermic mycosis fungoides following allogeneic HSCT to derive from a matched unrelated donor. ‘Transmission’ of donor-derived T-cell lymphoma and leukaemia via HSCT has been reported previously.^4^ ^9^ ^11^ In these instances, the sibling donors developed identical disease, but they demonstrated subclinical pathology at the time of diagnosis in the HSCT recipients. While these cases support the theory of a ‘malignant stem cell’ transferred within the allograft,^12^ temporal dissociations highlight the multifactorial aetiology of donor-derived secondary malignancies post-HSCT. Such risk factors are diverse but include donor genetics, total body irradiation, chronic GvHD and long-term immunosuppression.^3^ In this case, the patient had a higher risk of secondary malignancy by virtue of recurrent GvHD requiring prolonged and profound immunosuppression, which may have permitted immune evasion of an evolving malignant clone. With this in mind, it is interesting to note that the other two patients reported with primary CTCL post sibling donor allogeneic HSCT also received at least 1 year of oral immunosuppression with calcineurin inhibitors for GvHD exhibiting cutaneous manifestations.^5^ ^9^

Here, information related to the anonymous unrelated donor was understandably limited, but the relevant donor registry was informed of the potential risk of malignancy for the donor.

It is known that CTCL may present indolently,^11^ but the heterogenous presentation of this case, along with the requirement for ongoing GvHD therapy, complicated diagnosis. It may be postulated that ECP, originally developed for the treatment of erythrodermic CTCL,^13^ inhibited an earlier fulminant clinico-pathological presentation. Indeed, a review of histological examinations undertaken at the time of presumed cutaneous sarcoid suggested that in retrospect, the ‘cytologically bland’ lymphocytic infiltrates did have a similar folliculocentric pattern of infiltration as the later folliculotropic mycosis fungoides, and that the granulomatous response may have been due to a rupture reaction.

**Learning points**

- Survivorship following haematopoietic stem cell transplantation (HSCT) is of increasing importance as the safety of the procedures improves. Secondary malignancy is a devastating complication that contributes to the morbidity and mortality after HSCT.
- We present the first case of folliculotropic mycosis fungoides (stage IVA2) following HSCT to derive from an unrelated donor. A positive response to brentuximab is reported.
- We describe an evolving pathology with a multifactorial aetiology including intractable cutaneous graft versus host disease and long-term profound immunosuppression.
- Diagnosis required a high index of clinical suspicion and a cohesive multidisciplinary approach. This case highlights the vigilance required while monitoring patients following HSCT, and others on long-term immunosuppression for alternative diagnoses.
